# Stress Increases Ecological Risk of Glufosinate-Resistant Transgene Located on Alien Chromosomes in Hybrids Between Transgenic *Brassica napus* and Wild *Brassica juncea*

**DOI:** 10.3390/plants14040572

**Published:** 2025-02-13

**Authors:** Zicheng Shao, Lingling Dai, Longnan Liu, Sheng Qiang, Xiaoling Song

**Affiliations:** Weed Research Laboratory, College of Life Sciences, Nanjing Agricultural University, Nanjing 210095, China; 2019216027@stu.njau.edu.cn (Z.S.); 2022216037@stu.njau.edu.cn (L.D.); l18351007155@126.com (L.L.); wrl@njau.edu.cn (S.Q.)

**Keywords:** *Brassica juncea*, Brassica napus, gene silencing, methylation, stress, herbicide resistant

## Abstract

When glufosinate-resistant transgenic *Brassica napus* (transgene *PAT* located on C chromosome) were backcrossed with wild *Brassica juncea*, 50% of the progeny expressed *PAT* under favourable conditions. However, exposure to stress (drought, salt, flooding, and intraspecific competition) increased the proportion of plants expressing the *PAT* gene (r-e plants) by approximately 20% compared to those under unstressed conditions. In the self-pollinated progeny of the stressed plants, the proportion of r-e plants increased by a nearly 30% compared to that of the unstressed plants. Composite fitness was comparable between plants developed under drought stress at the seedling stage and those grown under favourable conditions. Abscisic acid (ABA) content and expression of the Repressor of Silencing 1 (*ROS1*) in leaves increased significantly after stress treatment in the progeny, with r-e plants exhibiting higher levels. Exogenous ABA treatment significantly up-regulated *ROS1* expression in progeny leaves, and the ABA treatment of seeds increased the survival of progeny exposed to glufosinate by 15%. Results suggest that increasing ABA under stress may enhance the demethylation of *PAT*’s promoter by promoting *ROS1* expression, thereby inhibiting transgene silencing of *PAT*, indicating that transgene located on the C chromosome of transgenic *B. napus* may pose a higher risk of gene flow to wild *B. juncea* under stress, especially drought stress.

## 1. Introduction

The stable inheritance and expression of herbicide-resistant transgenes in hybrids between transgenic crops, carrying them and their compatible relatives, determine the likelihood of transgene introgression [1,2,3,4,5]. With its characteristics of long flower season, long pollen flow distance, and long-term pollen viability, transgenic oilseed rape (*Brassica napus*, AACC; 2*n* = 38) may benefit its outcross with its relative, wild *Brassica juncea* (AABB; 2*n* = 36) [6]. The segregation of glufosinate resistance conferred by a transgene located on the C-chromosome of *B. napus* clearly departed from the expected Mendelian ratio. About half of the plants survived glufosinate selection in backcross progenies (BC1F3, BC1F4, and BC1F5) between wild *B*. *juncea* and glufosinate-resistant transgenic *B. napus* [6]. As nearly 50% of the progenies expressed *PAT* (glufosinate-resistant gene) normally (resistant-expression, r-e), the other half, also carrying the *PAT* gene, failed to do it (resistant-no-expression, r-n-e); i.e., gene silencing occurred, as verified by herbicide spraying and polymerase chain reaction (PCR) testing. We also found that DNA methylation of the *PAT* promoter sequence was a determining factor in transgene silencing [6]. However, these results were obtained under favourable planting conditions, without stress.

It is expected that transgenic plants grown in interaction with wild relatives would experience a diversity of environmental conditions. Wild *B. juncea*, the weedy relative of *B. napus*, is widely distributed in the wastelands and farmlands of the climatically variable Yangtze River and Yellow River basins and in northwest China [7], where plants are likely to be exposed to abiotic stresses, such as drought and flooding [8]. Therefore, determining whether these stresses influence the survival and fitness of progenies under glufosinate selection becomes relevant.

In plants, Repressor Of Silencing 1 (*ROS1*), Transcriptional Activator Demeter (*DME*) and Demeter-Like Protein (*DML*) play important roles in the regulation of demethylation, and *ROS1* mainly mediates the demethylation of the gene promoter region [9]. Gene silencing might be caused by methylation of the promoter sequence [6,10,11,12,13]. Therefore, *ROS1* might determine, in this case, the proportion of r-e plants under stress by mediating transgene promoter demethylation. *ROS1* mutants were hypersensitive to ABA during early seedling development and root elongation of Arabidopsis [14], which indicated a potential regulatory relationship between ABA and *ROS1*.

Stress regulates the content of the abscisic acid (ABA) in plants, which is widely recognized as a key hormone for regulating plant responses [15,16,17,18,19,20,21,22]. ABA binds to its receptor *PYR/PYL/RCAR*, a START domain protein, and the effector of ABA in plants will form a signal pathway network through the core signal of *PYL-PP2C-SnRK2* and other kinases to regulate plant physiology [23]. The ABA signaling pathway has been identified as a central regulator of abiotic stress response in plants, triggering major changes in gene expression and adaptive physiological responses [24]. Under stress conditions, plants synthesize ABA in various organs and initiate defense mechanisms, such as the regulation of stomatal aperture and expression of defense-related genes, conferring resistance to environmental stresses [25].

In this research, we aimed to (1) indicate genetic expression of *PAT* in backcross progenies between transgenic *B. napus* and wild *B. juncea* induced by stress; (2) elucidate the relationship between the DNA methylation of the transgene promoter sequence and gene silencing; (3) clarify the regulation of hormone ABA and demethylase *ROS1* in transgene silencing at the seedling stage; and (4) reveal the fitness-associated traits and composite fitness of backcross progenies under stress at the seedling stage. Above all, the results will not only provide a dimensional perspective on stress and gene silencing; they will also indicate the potential biosafety risk of herbicide-resistant transgenic *B. napus* under stress.

## 2. Results

### 2.1. Stress Increases the Proportion of Plants Expressing Glufosinate Resistance

**Figure 1:**
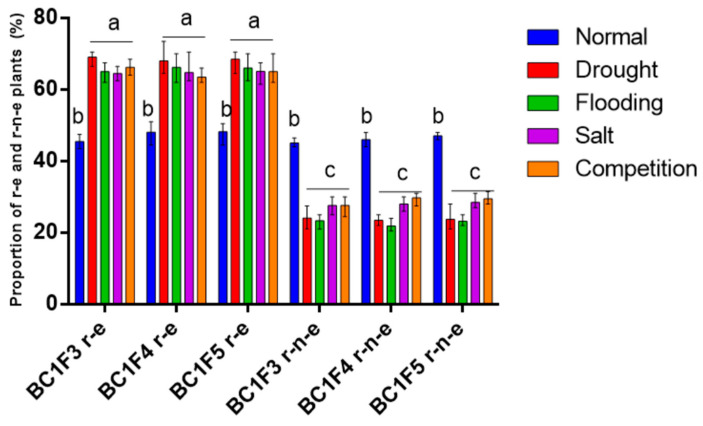
Proportion of r-e and r-n-e plants under stress conditions. Different letters represent significant differences at *p* < 0.05 (*n* = 4). The data are shown as the mean ± SEM.

The third, the fourth, and the fifth self-pollinated generations of backcross progenies between glufosinate-resistant *B. napus* and wild *B. juncea* (BC1F3, BC1F4, and BC1F5) were grown as seedlings under normal (unstressed) conditions and various stresses, including abiotic stresses, namely, drought, flooding, and salt stress; and biotic stress, namely, intraspecific competition. The proportion of r-e plants ranged from 43.5 to 50.5% under normal conditions but significantly increased to 64.5–73.5% under drought, 62.0–70.0% under flooding, 61.5–70.5% under salt stress, and 62.5–70.0% under competition (Figure 1). The proportion of r-e plants was similar across all stress treatments (Figure 1).

### 2.2. Prolonged Stress During the Seedling Stage Further Increases Glufosinate Resistance Expression

**Figure 2:**
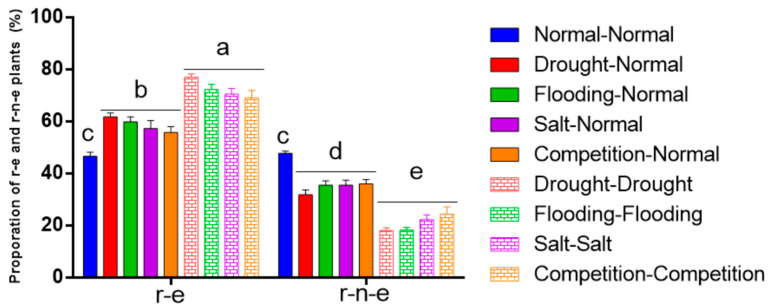
Proportion of r-e and r-n-e plants in BC1F3 and BC1F4 under different stress treatments. Different letters represent significant differences at *p* < 0.05 (*n* = 4). The data are shown as the mean ± SEM.

BC1F3-BC1F4 plants were subjected to three treatment regimens over two generations: Normal–Normal, Stress–Normal, and Stress–Stress (Figure 2). The highest proportion of r-e plants (65.0–79.5%) was observed in the Stress–Stress treatment, followed by the Stress–Normal treatment (51.5–64.5%), with the lowest proportion observed under Normal–Normal conditions (43.5–51.0%).

### 2.3. Methylation of the PAT Promoter Silences Glufosinate Resistance Under Normal and Stress Conditions

The *PAT* promoter was methylated at 4–8 sites in r-n-e plants but unmethylated in r-e plants (Table 1). The *PAT* region was methylated at 3–6 sites in both r-e and r-n-e plants. This methylation likely caused transgene silencing under both normal and stress conditions.

### 2.4. ABA Content and ROS1 Expression Are Up-Regulated by Stress

**Figure 3:**
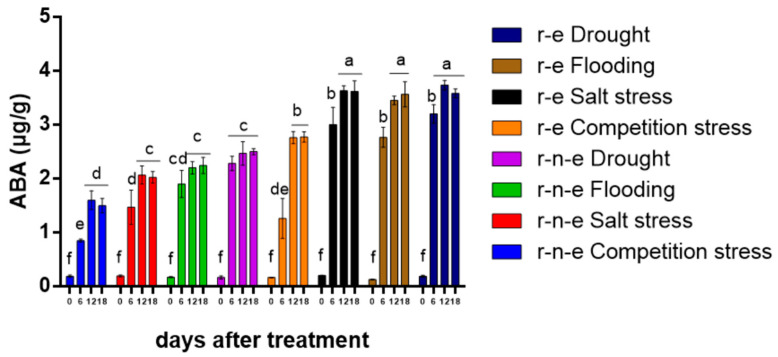
Content of ABA in r-e and r-n-e plants of BC1F4 under stress conditions. Different lowercase letters represent significant differences in all columns. The data are shown as the mean ± SEM. *p* < 0.05 (*n* = 4).

The results of BC1F4 were shown as example due to the highly consistent results of BC1F3-5. ABA content in plants of BC1F4 increased 5–10 fold in r-n-e and 7–15 fold in r-e plants after 6 days of stress (Figure 3). The increase was most pronounced under drought stress, followed by flooding, salt, and intraspecific competition. After 12 days, ABA content further increased by 7–10 fold in r-n-e and 12–18 fold in r-e plants, with no additional changes by day 18. Overall, ABA content was higher in r-e plants than in r-n-e plants.

**Figure 4:**
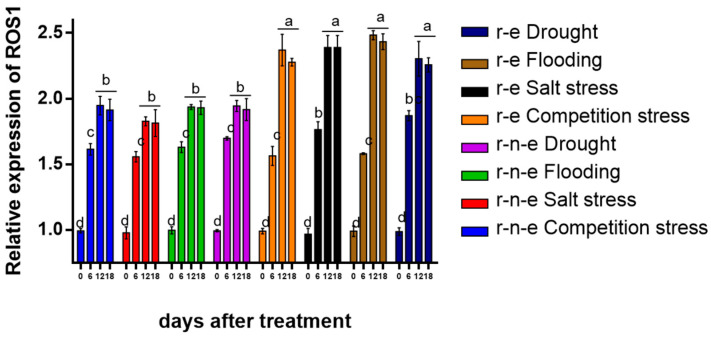
Relative expression of *ROS1* in r-e and r-n-e plants of BC1F4 under stress conditions compared with *HMG*. Different lowercase letters represent significant differences in the same column. The data are shown as the mean ± SEM. *p* < 0.05 (*n* = 4).

*ROS1* expression also increased significantly after 6 days of stress, with r-e plants showing a 1.5–1.9-fold increase (Figure 4). By day 12, *ROS1* expression increased further to 1.8–2.0 fold in r-n-e plants and 2.3–2.5 fold in r-e plants. No further change was observed after 18 days. *ROS1* expression was consistently higher in r-e plants than in r-n-e plants.

### 2.5. Expression of ROS1 in Progenies Was Up-Regulated by Exogenous ABA

**Figure 5:**
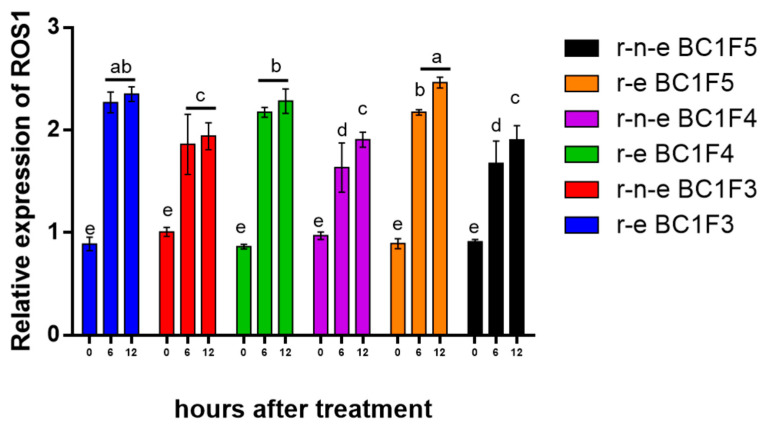
Relative expression of *ROS1* in r-e and r-n-e plants of BC1F3, BC1F4, and BC1F5 under ABA treatment. Different lowercase letters represent significant differences in all columns. The data are shown as the mean ± SEM. *p* < 0.05 (*n* = 4).

Exogenous ABA treatment (50 μM) of two-week old seedlings significantly upregulated *ROS1* expression in leaves of both r-e and r-n-e plants (Figure 5). After 6 and 12 h of ABA treatment, *ROS1* expression significantly increased by 1.3–2.3 fold and 1.7–2.5 fold, respectively. We posit that exogenous ABA reduces DNA methylation of the *PAT* promoter, thereby inhibiting gene silencing.

### 2.6. Exogenous ABA Treatment of Seeds Suppresses Transgene Silencing

Seed treatment with ABA at 50 µmol/L significantly increased the proportion of r-e plants among BC1F3, BC1F4, and BC1F5 progenies (Table 2). The positive effect of ABA was comparable to that of 5-azacytidine (5-AzaC), used as a positive control, indicating that ABA affects the phenotype early in plant growth.

### 2.7. Drought Stress at the Seedling Stage Does Not Affect Fitness-Associated Traits and Composite Fitness

**Figure 6:**
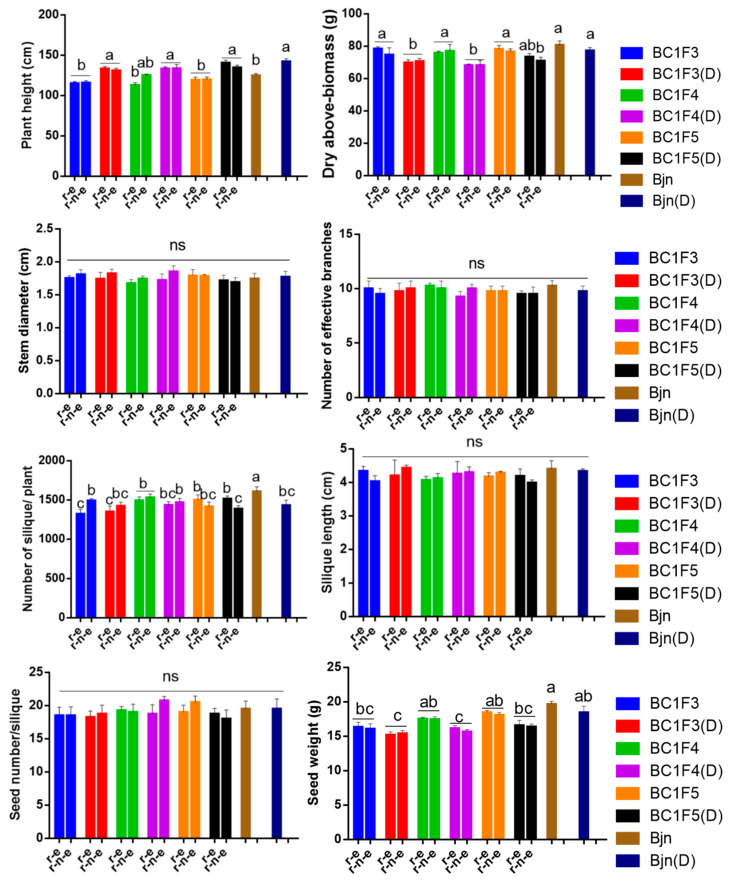
Fitness-associated traits of r-e and r-n-e plants in BC1F3-5 under drought stress and normal conditions at the seedling stage. Different lowercase letters represent significant differences in the same column. ‘ns’ represents no significant differences among columns. The data are shown as the mean ± SEM. *p* < 0.05 (*n* = 15).

Drought stress reduced the dry above-ground biomass and seed weight of most plants, except for BC1F5 and *B. juncea*, whose biomass was not affected (Figure 6). However, plant height was significantly higher in drought-stressed plants compared with those grown under favourable conditions. Composite fitness remained similar across treatments. The fitness-associated traits and composite fitness of r-e plants and r-n-e plants were comparable, suggesting that drought stress at the seedling stage may increase plant survival under glufosinate selection, thereby increasing potential ecological risks.

## 3. Discussion

We identified transgene silencing in the backcross progenies of transgenic *B. napus* and wild *B. juncea*, whose transgene was located on the C-chromosome, attributable to DNA methylation at the transgene promoter. Segregation of r-e and r-n-e individuals was observed in the self-pollinated progenies of the plants with silenced resistance genes [6]. These findings were obtained under favourable conditions but given the widespread distribution of wild *B. juncea* in the Yangtze and Yellow River basins, it is necessary to assess the risk of transgene flow in progenies exposed to different stresses.

We serendipitously discovered a case in which backcrossed progenies with insufficient soil moisture had a higher proportion of plants expressing the gene *PAT* after being treated with the herbicide compared with progenies with sufficient soil moisture, which indicated the potentially higher survival of progenies under stress, sparking our interest in elucidating this surprising response.

Our study focused on BC1F progenies grown under imposed-stress conditions (drought, flooding, salt stress, and intraspecific competition). We observed that the proportion of r-e plants subjected to stress was about 20% higher than that of plants grown under favourable conditions. Additionally, when these progenies were further subjected to stress at the seedling stage, the r-e proportion increased by an additional 10% compared with those developing under NC. Progenies from self-pollination under stress also had a higher r-e proportion. When these progenies face stress again during the seedling stage, the percentage rate of r-e plants significantly increased. This suggests that some r-n-e phenotypes converted to r-e, indicating an inhibition of gene silencing. As a result, these progenies had a higher survival rate under glufosinate selection, thus posing an elevated ecological risk. But how does this occur?

Previously, we suggested that the silencing of the glufosinate resistance gene, *PAT*, in progenies might be due to the methylation of its promoter region [6]. In this study, we also found that methylation sites on *PAT’S* promoter region induced gene silencing in progenies exposed to stresses. Therefore, removing the DNA methylation site at the *PAT* promoter could prevent silencing. The Repressor Of Silencing 1 (*ROS1*) is crucial in active demethylation regulation in plants, potentially inhibiting gene silencing by inducing demethylation at the gene promoter under stress [9,26]. Our research showed a significant increase in *ROS1* expression in progenies carrying the *PAT* gene after stress treatment. Concomitantly, r-e plants had a significantly higher *ROS1* expression compared with r-n-e plants under stress, suggesting that *ROS1* may mediate the demethylation of the *PAT* promoter, thereby preventing *PAT* silencing. Since ABA content and *ROS1* expression levels increased significantly under stress, and ABA treatment of leaves and seeds also elevated *ROS1* expression and the r-e proportion, there is likely a regulatory relationship between ABA and *ROS1* under stress.

Plants might engage *ROS1*-mediated DNA demethylation on ABA-inducible genes for ABA responses in *Arabidopsis thaliana* [14]. This implies that stress activates the ABA signaling pathway, and *ROS1* may enhance ABA’s role in stress resistance by demethylating ABA-inducible genes. *ROS1* also increased the expression of the ABA pathway-related genes, *ACO3* and *GSTF14*, in *A. thaliana* by demethylating their promoters, indicating that *ROS1* may be involved in ABA-related molecular mechanisms under stress [27].

Our study demonstrates a positive regulatory relationship between ABA and *ROS1*, though the precise molecular mechanisms require further investigation using suitable mutants. The increased levels of ABA and *ROS1* in r-e plants compared to r-n-e plants under stress suggest that r-e plants are more sensitive to stress. We speculate that ABA and *ROS1* may play a role in inhibiting gene silencing.

DNA methylation and demethylation in plants are typically influenced by environmental factors and play important roles in coordinating plant growth and stress responses [28,29]. Abiotic stress induces active demethylation and raises demethylase expression [9,30,31]. In our study, gene silencing was attributed to methylation at the *PAT* promoter, which might be related to heterochromatinization in the insertion region of resistance genes during cell division [32], leading us to focus on *ROS1* for its role in inducing demethylation. Other demethylases, such as Transcriptional Activator Demeter (*DME*) and Demeter-Like Protein (*DML*), not considered in our study, deserve further research since they may also mediate active demethylation.

Stress influences the expression of transcription factors and functional proteins in plant gene networks [33]. However, the complete inhibition of gene silencing has not been reported. We found that imposing stress on seedlings increased the proportion of r-e plants. However, applying stress at other growth stages (bolting, flowering, and podding) did not affect the proportion or plant phenotype. The exact timing of gene silencing remains uncertain as glufosinate resistance could not be determined until 20 days after sowing. It is possible that the *PAT* gene is more unstable and susceptible to the environmental changes, making it more prone to silencing inhibition at the seedling stage under stress by a mechanism to be determined.

Progenies of BC1F3, BC1F4, BC1F5, and wild *B. juncea* subjected to drought stress at the seedling stage had similar fitness-associated traits and composite fitness. All treatments groups showed an over-98% survival rate before transplanting on the 9th of November. However, we attempted to quantify traits in plants subjected to flooding, salt stress, and competition but high mortality among treated seedlings after the day of transplanting precluded this. In practice, drought stress in autumn may enhance the survival of GR progenies under herbicide selection, potentially increasing its ecological risks.

We propose a gene silencing inhibition mechanism wherein stress induces demethylation at the *PAT* promoter by up-regulating ABA levels and mediating *ROS1* expression. This ultimately increases the proportion of individuals in the population that can withstand the application of the non-selective herbicide glufosinate. However, several questions remain: (1) Why does gene silencing inhibition occur only during the seedling stage? (2) How is the probability of gene silencing after stress inherited in subsequent self-pollinated generations? (3) How does ABA regulate *ROS1* expression through molecular signal pathways? While we discovered that stress disrupts the “dynamic equilibrium” of gene expression, it is unclear whether similar cases exist in other plants. Importantly, the inhibition of gene silencing by stress presents a new perspective for assessing the biosafety of herbicide-resistant *B. napus*. Given the inevitable occurrence of extreme weather events in regions where wild *B. juncea* is distributed, biosafety assessments of transgenic plants under stress conditions are crucial. The increased proportion of *PAT*-expressing plants after stress suggests that glufosinate-resistant *B. napus* may pose higher ecological risk than under favourable conditions, warranting greater attention in agricultural production.

## 4. Materials and Methods

### 4.1. Plant Materials

**Figure 7:**
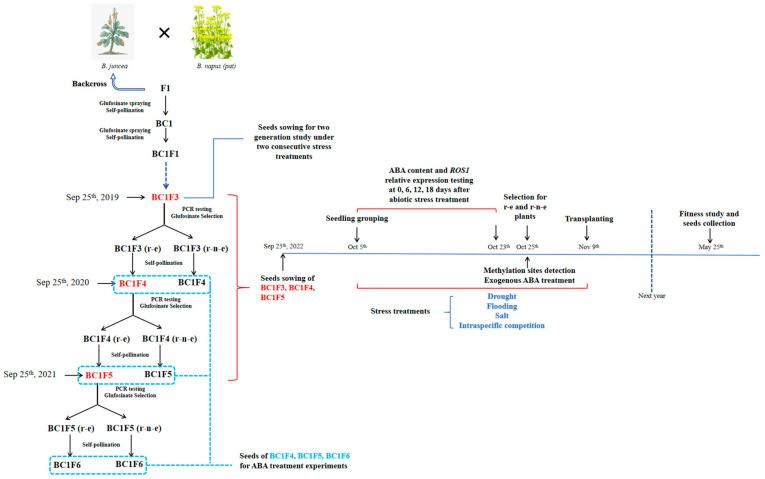
Segregation map of backcrossed progenies’ herbicide-resistant traits and the research schedule.

The transgenic oilseed rape (*B. napus* L.; genome, AACC; diploid chromosome number, 2*n* = 38) used in this study carriers the *PAT* transgene that confers glufosinate resistance (event HCN28) in its C8 chromosome. This transgenic line was provided by Dr. Wenming Zhang from Agriculture and Agri-Food Canada (Ottawa Research and Development Centre) and was originally produced by transforming the spring-type *B. napus* cv. AC Excel. Wild *B. juncea* seedlings (genome AABB; 2*n* = 36) were collected from a winter wheat field in Jiangpu, Nanjing City, China. The seedlings were identified using SSR markers, as described by Sun et al. (2018) [7]. The crossing scheme of hybridization and backcrossing between wild *B. juncea* and GR *B. napus* is illustrated in Figure 7. Seeds of wild *B. juncea* were stored at 4 °C until use. GR BC1F2 seedlings, derived from crosses between wild *B. juncea* and GR *B. napus*, were obtained according to the methods described by Song et al. (2021) [34]. Among the GR BC1F2 plants, the ten that exhibited the highest seed fertility were selected for self-pollination, and BC1F3 seeds were collected in 2019. From each of the 10 mother plants, at least 250 self-pollinated BC1F3 seeds (a total of 2500 seeds) were randomly selected for planting. For control, 100 seeds of wild *B. juncea* were also planted. Seeds were sown on 25 September 2019, and the plants allowed them to grow until 25 May 2020. Following the selection for resistance-not-expressed (r-n-e) and resistance-expressed (r-e) plants in the BC1F3 generation, as described by Shao et al. (2022) [6], the plants were transplanted and self-pollinated to produce BC1F4, which were stored as before. BC1F5 seeds were obtained by following the same procedures as with BC1F4. The crossing scheme of BC1F3, BC1F4, and BC1F5 generations is presented in Figure 7.

### 4.2. Biochemical Reagents, Services, and Instruments

Biochemical reagents were obtained from the providers indicated in parenthesis: Glufosinate (18% glufosinate-ammonium, AS, Baosteel, Bayer Crop Sciences, Leverkusen, Germany); BU Taq 2× Master PCR mix (Yangzhou Baosheng Biotechnology Co., Ltd., Yangzhou, China); Enzyme-linked Immunosorbent Assay (ELISA) Kit (Nanjing Meilin Xuehai Biotechnology Co., Ltd., Nanjing, China); Cloning vector PMDTM 19-T (Takara Biomedical Technology (Beijing) Co., Ltd., Beijing, China); *E. coli* DH5α competent cells (Takara Biomedical Technology (Beijing) Co., Ltd., Beijing, China); Taq enzyme and T4 DNA Ligase (Takara Biomedical Technology (Beijing) Co., Ltd., Beijing, China); EZ DNA Methylation-GoldTM Kit (ZYMO Research Corporation, Irvine, CA, USA); PCR Master mix (Takara Biomedical Technology (Beijing) Co., Ltd., Beijing, China); IPTG (Takara Biomedical Technology (Beijing) Co., Ltd., Beijing, China); ampicillin (Macklin Inc., Shanghai, China); 5-azacitidine (Sigma-Aldrich, Shanghai, China).

DNA sequencing was contracted to Sangon Biotech Co., Ltd., Shanghai, China.

The following instruments were used in conducting the study: Constant Temperature Incubator (XMTD-8222, Jinghong Precision Technology Co., Ltd., Shenzhen, China); polymerase chain reaction (PCR) machine (Takara Biomedical Technology (Beijing) Co., Ltd., Beijing, China); and DYY-8B Electrophoresis Apparatus (Nanda Biotechnology Inc., Nanjing, China.).

### 4.3. Planting and Growing Conditions of BC1F3, BC1F4, and BC1F5

Seeds were sown in 6 cm diameter pots filled with a mixture of garden soil and peat in a 1:1 volume ratio. Emerged seedlings were grown under natural light, with a photoperiod ranging from 10 to 13 h. Air temperatures fluctuated between 5 and 15 °C from transplanting to bolting, and between 10 and 28 °C from bolting to harvest. Each pot contained a single plant, and the pots were placed 10 cm apart in the greenhouse. All cultural practices up to harvest followed the procedures described by Song et al. (2010) [35].

### 4.4. Responses of BC1F3, BC1F4, and BC1F5 Under Stress

#### 4.4.1. Testing of Soil Field Capacity (FC)

Soil samples were collected, contained in an aluminum box, and placed in a constant temperature incubator (XMTD-8222, Jinghong Precision Technology Co., Ltd.) at 105 °C for 24 h to completely evaporate the moisture in the soil (until the box maintained constant weight). The box was weighted according to an analytical balance. We collected the soil with a cutting ring and placed it into a flat bottomed tray. We added water into the tray and kept the water surface 1–2 mm lower than the upper edge of cutting ring. We soaked the cutting ring for 24 h, and we removed the water-saturated cutting ring from the tray, added the top lid, and removed the perforated bottom lid. We placed the cutting ring on another cutting ring, which was covered by filter paper and contained the dried soil sample. We used a heavy object (2 kg) to compact the soil and used two cutting rings in close contact. After 8 h of water infiltration, we took 20–30 g of upper soil sample from the upper ring cutter and placed it in a weighted aluminum box (M0) and weighed the total mass (M1). We placed the box in the constant-temperature incubator at 105 °C for 24 h to completely evaporate the moisture in the soil (until the box maintained a constant weight) and then weighed it (M2). The formula for calculating soil field capacity (FC) is as follows:

FC = (M1 − M2)/(M2 − M0) × 100%

When the soil moisture is ≤ 20% FC, the plant survival of plants is too low to support subsequent experiments; if the soil moisture was ≥ 40% FC, there would be no significant difference in the proportion of r-e plants compared with plants under favourable treatment. So, we chose 30% FC of soil moisture as a suitable drought stress treatment.

#### 4.4.2. Soil Moisture Testing

We put the plastic pots containing soil into the electric furnace at 105 °C for 24 h to evaporate the moisture in the soil. Then, we weighed the pots and recorded the results (m0). In the growth of plants in the seedling stage, we constantly weighed the pots (m) and analyzed the soil moisture of the pots by testing the differences in pot weight compared with m0.

Soil moisture = (m − m0)/m0 × 100%

#### 4.4.3. Response to Drought Stress

Before sowing BC1F3, BC1F4, and BC1F5 seeds, the field capacity (FC) of the growing medium (garden soil and peat mixture at a 1:1 volume ratio) was determined as described above. The normal unstressed condition (NC) group used a medium at 50% FC, while the drought stress condition used medium at 30% FC based on the preliminary results detailed above. Two hundred seedlings of each BC1F3, BC1F4, and BC1F5 were grown under drought conditions, and an equal number were grown under NC. Meanwhile, another 100 of each BC1F3, BC1F4, and BC1F5 were grown under drought conditions for plants weighing. After sowing, 3 BC1F3, BC1F4, and BC1F5 were randomly selected at 10:00 am every day, removed from the pot, and cleaned to remove the medium attached to the roots before weighing them. Plant weight was used for calculating and controlling medium moisture during daily weighing. The medium moisture was maintained at 50% FC for 10 days by weighing daily and re-watering the pots at 10:00 a.m. From then, until 30 days after sowing (DAS), plants exposed to stress were grown at 30% FC, while plants not subjected to moisture stress remained at 50% FC.

#### 4.4.4. Response to Flooding Stress

BC1F3, BC1F4, and BC1F5 plants were cultured for 10 days under normal unstressed conditions then transferred into 40 cm × 70 cm × 10 cm clean plastic trays filled with water to maintain a 5 cm water level. This flood condition was maintained for 30 days, with water replenished every 72 h. Two hundred seedlings of each BC1F3, BC1F4, and BC1F5 were grown under flooding conditions, while another 200 seedlings were grown under NC.

#### 4.4.5. Response to Salt Stress

Ten-day old seedlings of BC1F3, BC1F4, and BC1F5 growing under normal unstressed conditions were then watered with a 0.5 mM concentration of NaCl/ddH_2_O solution. The medium moisture was kept at 50% FC (similar to favourable conditions) via the weighting of pots for 30 days. Two hundred seedlings of each BC1F3, BC1F4, and BC1F5 were grown under salt stress conditions, while an equal number were grown NC.

#### 4.4.6. Response to Intraspecific Competition Stress

Ten seeds each of BC1F3, BC1F4, and BC1F5 were evenly sown in 60 pots, with each seed occupying only 10% of the planting space of NC group. The pots were maintained under normal unstressed conditions for 30 days. Two hundred seedlings of each BC1F3, BC1F4, and BC1F5 were individually grown under NC. Other procedures followed those used in the flooding stress study.

### 4.5. Measurement of ABA Content and ROS1 Relative Expression

At 10 DAS, 200 uniform-size seedlings of each BC1F3, BC1F4, and BC1F5 were selected for stress treatments, as described in Section 4.4. An equal number of seedlings were grown under NC. Individual seedlings from each generation (BC1F3, BC1F4, and BC1F5) were identified by number (1–200) and divided into four equal groups: No. 1–50, No. 51–100, No. 101–150, and No. 151–200. Twenty plants from each group (a total of 80 plants per generation) were randomly selected for the collection of the first, second, third, and fourth leaves at 10, 16, 22, and 28 DAS (equivalent to 0, 6, 12, and 18 days after drought stress treatment) to determine the ABA content by using ELISA and for *ROS1* relative expression by qRCR testing.

### 4.6. Selection of r-e and r-n-e Plants

The top leaves of 30-day old seedlings from the different backcross progenies were collected, frozen in liquid nitrogen, and then stored at −80 °C. Based on PCR testing for the *PAT* gene (primers provided in Table S1) and the glufosinate screening methods described by Shao et al. (2022) [6], the progenies were characterized into three types: resistance-expressed (r-e), for plants carrying and expressing the *PAT* gene; resistance-not-expressed (r-n-e), for plants carrying but not expressing the *PAT* gene; and non-expressing (n) for plants not carrying the *PAT* gene. Proportion of r-e and r-n-e plants was recorded.

### 4.7. DNA Methylation Site Detection

A total of 20 r-e plants and 20 r-n-e plants from each BC1F3, BC1F4, and BC1F5 generations were randomly chosen for DNA methylation site detection, as described in Supplementary Methods Section S3. If cytosine (C) is methylated, it will not be converted to uracil (U) under sulfite treatment and thus remains as cytosine (C) in the gene sequence. The transformants were commercially sequenced. Using the analysis software Bioedit V7.0.9, the sequences were compared with the original sequence of the transgene (CaMV35s and *PAT* gene) to identify the location and number of methylated cytosine (C) sites.

### 4.8. Fitness Test

A total of 15 r-e plants and 15 r-n-e plants form BC1F3, BC1F4, and BC1F5, along with 15 wild *B. juncea* plants, were grown under normal unstressed and drought stress conditions (a total 105 plants). These plants were individually transplanted into pots (25 cm diameter) containing the same growth medium described previously after 35 days of drought stress (45 DAS). After transplanting, the plants were grown under normal unstressed conditions. On the 25th of May of the following year, traits associated with both vegetative and reproductive growth were measured. Vegetative fitness traits included plant height, stem diameter, number of effective branches, and dry above-ground biomass. Reproductive stage fitness traits included the number of siliques per plant, silique length, seed number per silique, and seed weight (see Table S9 for specific measurement methods). Fitness was estimated as described in Section S5.2 of Supplementary Methods and comparisons were made between drought stress and normal unstressed conditions and between r-e and r-n-e plants.

### 4.9. Two Generation Study Under Two Consecutive Stress Treatments

Seed selection and planting methods for BC1F3 followed those described in Section 4.4. The proportion of r-e plants under drought, flooding, salt and intraspecific competition stress conditions was recorded, and the seedlings were transplanted as in Section 4.4. Five plants from each of the r-e and r-n-e groups were randomly selected for self-pollination. After self-pollination, seeds of these plants were collected and mixed randomly. A hundred seeds from r-e and r-n-e plants (total 200 seeds) from the different treatment groups were randomly selected and planted the following year, treated similarly to BC1F3 (normal unstressed and stressed conditions) at the seedling stage.

**Figure 8:**
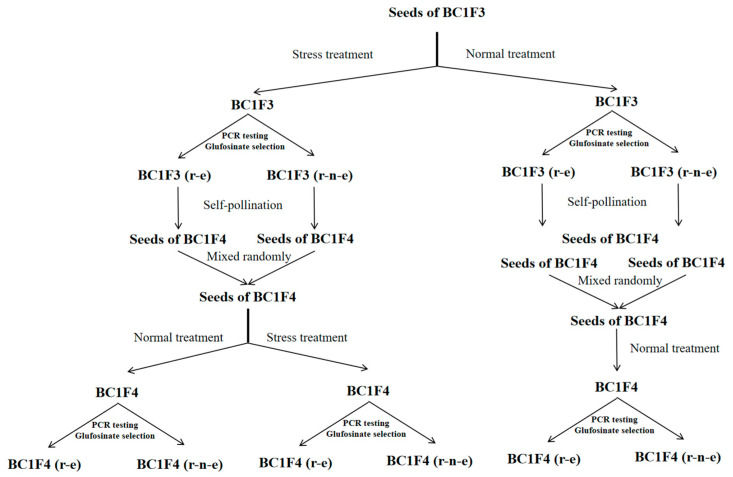
Schedule of two-generation study under two consecutive stress treatments.

Progenies treated under normal unstressed conditions in both generations were designated as the Normal–Normal group (Figure 8). Progenies treated under stressed conditions and then under normal unstressed conditions in the following generation were labeled as Drought–Normal, Flooding–Normal, Salt–Normal, and Competition–Normal groups (Figure 8). Progenies treated under stress conditions in both generations were designated as Drought–Drought, Flooding–Flooding, Salt–Salt, and Competition–Competition groups (Figure 8). In the second generation, the proportion of r-e plants was recorded for comparative analysis. Differences of proportion of r-e plants among the treatment combinations over two generations were analyzed (Figure 8). A map of the two consecutive generations is presented in Figure 8.

### 4.10. Responses of Progenies to ABA

#### 4.10.1. Response to Exogenous ABA Treatment

Seeds from BC1F3, BC1F4, and BC1F5 were sown, and seedlings were cultured under normal unstressed conditions (Normal group). After the selection of the r-e and r-n-e plants (30 days after sowing), 12 seedlings were randomly selected from the r-e and r-n-e groups in each generation. ABA/ddH_2_0 solution (50 µmol/L) was applied to the top three leaves of each seedling using a brush. Treated and control leaves were sampled at 0, 6, and 12 h after ABA application for *ROS1* relative expression analysis.

#### 4.10.2. Response to ABA Treated to Seeds

Ten r-e and r-n-e plants from each BC1F3, BC1F4, and BC1F5 generation were selected, and 10 seeds were randomly taken from each plant (as marked with a blue rectangle in Figure 7). A total of 200 seeds of each of the generations were placed in petri dishes containing either ABA/ddH_2_0 solution (50 µmol/L) or ddH_2_O alone as a negative control at room temperature (20 ± 5 °C) for 6 h. An additional 200 seeds from each generation were incubated with 5-AzaC, a nucleoside-based DNA methyltransferase inhibitor that induces global DNA hypomethylation and gene reactivation [36,37]. Petri dishes lined with filter paper moistened with 5-AzaC (250 µmol/L) were randomly placed in a 4 °C cold chamber to temporarily inhibit seed germination; the saturated filter paper was replaced daily for 5 consecutive days. Seeds treated with 5-AzaC were prepared 5 days before the ABA treatment to synchronize the experiment’s end time so that seeds treated with 5-Azac could germinate simultaneously with seeds treated with ABA and ddH_2_O. Seeds were then sown, and seedlings were cultured as described in Section 4.3. After the selection of r-e and r-n-e plants, as described in Section 4.4, the proportion of r-e plants was calculated and compared among ABA, ddH_2_O (negative control), and 5-AzaC (positive control) treatments.

### 4.11. Statistical Analyses

All data were initially subjected to a two-way analysis of variance (ANOVA), and means were separated using Tukey’s test at *p* < 0.05. All statistical analyses were conducted using SPSS 20.0 statistical software (International Business Machines Corporation, New York, NY, USA). All gene sequence analyses and primer designs were performed using Primer Premier 6.0 (Primer, San Francisco, CA, USA).

## 5. Conclusions

Stress significantly increased the proportion of backcross progenies that expressed *PAT* between glufosinate-resistant *B. napus* and wild *B. juncea*, with the increase inheritable to the next self-pollinated generation. The ABA content and relative expression of *ROS1* in progeny leaves were significantly elevated under stress at seedling stage. Exogenous ABA significantly upregulated *ROS1* expression in leaves. Exogenous ABA treatment of seeds also significantly increased the proportion of progenies expressing *PAT*. Drought stress at the seedling stage did not significantly influence fitness-associated traits and composite fitness of progenies. The results indicate that stress inhibits gene silencing through ABA-*ROS1* regulation and might up-regulate the survival of progenies under glufosinate selection, thus increasing the gene flow risk of transgenic oilseed rape. This study provides a theoretical and experimental basis for considering potential ecological risks under stress conditions while designing the insertion of exogenous genes into the low-risk C chromosome.

## Figures and Tables

**Table 1:** Number of methylation sites of transgenes (promoter and *PAT* gene) in backcross generations of glufosinate-resistant transgenic *B. napus* and wild *B. juncea*.

Backcross Progenies	Stress Treatment	Resistant-Expressed		Resistant-Not-Expressed	
		Promoter Region	*PAT* Region	Promoter Region	*PAT* Region
BC1F3	Normal	0	4	6	3
	Drought	0	5	5	4
	Flooding	0	4	8	4
	Salt	0	4	7	6
	Competition	0	3	6	4
BC1F4	Normal	0	4	6	5
	Drought	0	4	7	5
	Flooding	0	5	6	5
	Salt	0	4	4	3
	Competition	0	5	6	5
BC1F5	Normal	0	5	6	4
	Drought	0	3	5	4
	Flooding	0	6	8	5
	Salt	0	4	6	5
	Competition	0	4	5	5

**Table 2:** Proportion of r-e plants under seeds treatment by ABA and 5-AzaC.

Backcross Progenies	Seed Treatment	Number of Plants	Number of Survived Plants	Proportion of r-e Plants (%)
BC1F3	No	200	93.00 ± 2.08	46.50 ± 1.04 c
BC1F4	No	200	104 ± 5.57	52.00 ± 2.78 c
BC1F5	No	200	96.33 ± 3.67	48.17 ± 1.83 c
BC1F3	ABA	200	114.33 ± 2.60	57.17 ± 1.30 b
BC1F4	ABA	200	128.67 ± 4.67	64.34 ± 2.33 b
BC1F5	ABA	200	127.33 ± 4.67	63.67 ± 2.33 b
BC1F3	5-AzaC	200	133.33 ± 3.33	66.67 ± 1.67 a
BC1F4	5-AzaC	200	145.67 ± 2.96	72.88 ± 1.48 a
BC1F5	5-AzaC	200	131.00 ± 3.22	65.5 ± 1.61 a

ABA treatment groups showed a higher survival rate compared with the normal treatment group. Different lowercase letters represent significant differences in the generation. The data are shown as the mean ± SEM. *p* < 0.05 (*n* = 3).

## Data Availability

The data are available upon request from the corresponding author.

## Supplementary Materials

The following supporting information can be downloaded at https://www.mdpi.com/article/10.3390/plants14040572/s1: Table S1: DNA PCR Primers for *PAT* gene; Table S2: The system of polymerase chain reaction for *PAT* gene; Table S3: Preparation for the reverse transcription master mix of *ROS1* genes in the backcross generation between wild *Brasscia juncea* and transgenic glufosinate-resistant *B. napus*; Table S4: Reaction for the reverse transcription master mix of *ROS1* in the backcross generation between wild *B. juncea* and transgenic glufosinate-resistant *B. napus*; Table S5: qPCR primers for *ROS1* and HMG genes in progenies of the first backcross generations between glufosinate-resistant transgenic *B. napus* and wild *B. juncea*; Table S6: qPCR amplification reaction system of *ROS1* genes in the backcross generation between wild *B. juncea* and transgenic glufosinate-resistant *B. napus*; Table S7: The primers for DNA methylation PCR of transformant promoter (CaMV35S) and *PAT* gene; Table S8: The system for PCR of transformant promoter (CaMV35S) and *PAT* gene; Table S9: Traits measured for relative fitness estimation at two growth stages. Ref. [38] was cited in the Supplementary Materials.
