# Supplementary material for: Stress Increases Ecological Risk of Glufosinate-Resistant Transgene Located on Alien Chromosomes in Hybrids Between Transgenic Brassica napus and Wild Brassica juncea
Source: Plants (Basel). 2025 Feb 13;14(4):572. doi: 10.3390/plants14040572 (PMC11859238; doi:10.3390/plants14040572)
Supplement: Supplementary file 1 [file plants-14-00572-s001.zip › Sup Materials.pdf]

Supplementary Methods

1. Drought stress treatment

1.1 Testing of soil field capacity (FC)

Soil sample was collected, contained in an aluminum box and placed in a constant temperature incubator (XMTD-8222, Jinghong Precision Technology Co., Ltd) at 105°C for 24 hours to completely evaporate the moisture in the soil (until the box maintained constant weight). Box was weighted by an analytical balance. Collected the soil by a cutting ring and placed into a flat bottomed tray. Added water into the tray and kept the water surface 1-2 mm lower than the upper edge of cutting ring. Soaked the cutting ring for 24 hours. Removed the water-saturated cutting ring from the tray, added the top lid and removed the perforated bottom lid. Placed the cutting ring on another cutting ring which was covered by filter paper and contained dried soil sample. Used a heavy object (2 kg) to compact and made two cutting rings in close contact. After 8 hours of water infiltration, took 20-30 grams of upper soil sample from the upper ring cutter and placed it in a weighted aluminum box (M0) and weighted the total mass of them (M1). Placed the box in the constant temperature incubator at 105°C for 24 hours to completely evaporate the moisture in the soil (until the box maintained constant weight) and then weighted (M2). The formula for calculating soil field capacity (FC) is as follows:

$$FC=(M1-M2)/(M2-M0)\times 100\%$$

When the soil moisture≤20% FC , the plant survival of plants was too low to support subsequent experiments; When the soil moisture ≥ 40% FC, there would be no significant difference in the proportion of r-e plants compared with plants under favourable treatment. So we chose 30%FC of soil moisture for suitable drought stress treatment.

1.2 Soil moisture testing

Put the plastic pots contained soil into the electric furnace at 105°C for 24 hours to

evaporate the moisture in the soil. Then weighted the pots and recorded (m0). In the growth of plants in seedling stage, constantly weighted the pots (m) and analyzed the the soil moisture of the pots by testing the differences of pots weight compared with m0.

$$\text{Soil moisture}=(m-m0)/m0\times 100\%$$

2 Extraction of DNA template

Extract the total DNA of the material by SDS method:

① Weighed 0.2 g of the leaves as the test material, froze and grinded them into powder with liquid nitrogen, and transferred them to about 0.3 ml of a 2.0 ml centrifuge tube;

② Added 600 μL of 65 °C preheated extraction solution, mix well, and then bathed at 65 °C for 30 minutes, during which shook for 3-4 times until the sample solution is dark green;

③ Added 150 mL NaAc (1/4 of the extract) and shook well;

④ Added 600 mL of chloroform: isoamyl alcohol (24:1), shook well, centrifuged at 12000 r/min for 5 min, and transferred 500 μL of supernatant to another 2.0ml centrifuge tube

⑤ Added anhydrous ethanol 1000 μL (twice the volume of supernatant), evenly mixed, 12000 r/min, centrifuged for 5 min;

⑥ Discarded the supernatant, added 400 mL volume fraction of 70% ethanol, mixed it slightly (or place it for about 10min), 12000 r/min, centrifuged for 2-3 minutes, poured the alcohol and dry it at room temperature;

⑦ Added 200-400 μL TE or double distilled water (1μL RNA enzyme can be added) into the centrifuge tube containing DNA, and gently flicked it with hand to dissolve the sediment- Store at 20 °C.

See Table S2 for PCR system

Took 3 μL PCR products were detected by agarose gel electrophoresis.

3 Testing of *ROS1* relative expression

After 30 days of seedlings, top first leaves of different backcross progenies were taken and then frozen in liquid nitrogen and stored at -80℃. RNA extraction was carried out according to the instructions of the RNA Extraction Kit Biospin Plant Total RNA Extraction Kit from Bioflux. Preparation of the reverse transcription master mix of *ROS1* genes are shown in Table S3. Reaction system of the reverse transcription master mix of *ROS1* genes were shown in Table S4. Two primers of *ROS1* designd by software of Primer-Primer 5 which were shown in Table S5.qPCR amplification reaction system of *ROS1* genes were shown in Table S6.

3.1 RNA extraction

RNA extraction was carried out according to the instructions of the RNA Extraction Kit biospin plant total RNA extraction kit of bioflux. The specific operating steps are as follows:

1) Reagent preparation: add in lysis before operation β- Mercaptoethanol to a final concentration of 5%. Take 500 μL lysis, added to a 1.5ml centrifuge tube, and 50 μL plantaid, standby.

2) Sample pretreatment: after grinding an appropriate amount of fine powder of plant tissue in liquid nitrogen, weigh an appropriate amount of fine powder and put it into the above 1.5ml centrifuge tube equipped with lysis and plantaid, immediately shake it violently until there is no obvious granule, and leave it at room temperature for 5min.

3) The lysates were centrifuged at 1300rpm for 10min, and the fragments that could not be lysed and plantaid bound with polysaccharide polyphenols were precipitated, and 420 μ L lysate supernatant was transferred to a new 1.5ml centrifuge tube.

4) Accurately estimate the supernatant volume of the lysate, add 0.5 times the volume of absolute ethanol, blow and mix immediately, and do not centrifuge.

5) The mixture was sucked into the spin column, the spin column was sleeved with a centrifuge tube, and centrifuged at 1300rpm for 1min

6) Discard the liquid in the outer sleeve and add 600 to the spin column μ L PG buffer, stand at room temperature for 30s, centrifuge at 12000rpm for 30s, and discard the liquid in the liquid receiving pipe.

7) Add 600 to spin column μL wash buffer, centrifuge at 12000rpm for 30s, and discard

the liquid in the liquid receiving pipe. Join 600 μL wash buffer to wash again.

8) Then the empty column was centrifuged at 12000rpm for 1min, and the wash buffer was removed as much as possible to prevent the residual ethanol in the wash buffer from inhibiting the downstream reaction.

9) Move the spin column into a new 1.5ml centrifuge tube, and add the regeneration buffer 50 in the center of the membrane μ l. Total RNA was obtained by standing at room temperature for 1min.

Since RNA is extremely unstable and easy to decompose, the extracted RNA is stored in a - 80 °C refrigerator.

3.2 cDNA synthesis

According to the reverse transcriptase primescript ™ RT reagent kit with gDNA eraser (perfect real time) (Takara company) instructions to prepare reaction solution:

1. Prepare the reaction mixture on ice according to Table S3. Prepare the mixture and pack it into the reaction tube, and finally add RNA sample.
2. The above reaction solution was treated at 42 °C for 2 min.
3. The reaction solution shall be prepared according to Table S4. The mixed solution shall be prepared according to the amount of reaction number + 2, and then each tube shall be divided into 10 μL. After mixing, the reverse transcription reaction shall be carried out immediately.
4. The above reaction solution was treated at 37 °C for 15min and 85 °C for 5S.
5. Store the cDNA at - 20 °C for standby.
6. qPCR primers and amplification reaction system of *ROS1* genes was set according to Table S5&6.

4 Methylation site detection

Twelve plants were randomly selected as a group from r-e and r-n-e plants of BC1F3, BC1F4 and BC1F5. Totally 72 leave samples were collected for methylation site detection. DNA methylation was performed by bisulfite treatment according to the instructions of Methyldetectortm Bisulfite Modification kit.

4.1 DNA sulfite treatment

DNA methylation was performed by bisμLfite treatment according to the instructions of methyldetectortm bisulfite modification kit. The amount of DNA was 500 pg^{-2} μg. The optimum concentration is 200-500ng. The experimental process is as follows:

(1) Buffer preparation

① Conversion Buffer

Take 1 tube of CT conversion reagent and add:

ddH_2O 900 μL

M-Dilution Buffer 300 μL

M-Denaturation Reagent 175 μL

Shake it on the shaking table for 10 minutes at room temperature. It should be used immediately after preparation, or it can be stored at - 20 °C for one week.

① Preparation of m-wash-buffer

Add 24 ml of 100% ethanol to the m-wash buffer to configure a usable wash buffer.

(2) Conversion reaction

① Prepare CT conversion buffer solution.

② In the order of 200 μL Add the following reagents to the PCR tube:

CT Conversion Buffer 130 μL

DNA 20 μL

If the volume of the DNA sample is less than 20 ml, use water to make up for the difference and mix the sample.

③ Place the sample in the PCR instrument, and the procedure is as follows:

98°C 10min

64°C 2.5h

Store the product at 4 ° C (up to 20 h).

(3) Upper column desulfurization and DNA purification

① Add 600μL m-binding buffer to the zymo spin IC column and place the column in the collection tube.

② Add the obtained PCR product to the zymo spin IC column with m-binding buffer and mix the samples.

② Centrifugal (≥ 1000 × g) 30s, pour the waste liquid.

③ Add all the reaction liquid into the collection tube, centrifuge at 10000 rpm for 30s.

⑤ Transfer 200 μ L DNA wash buffer into the tube, 10000 rpm, and centrifuged for 30s.

⑥ Add 200 μL m-wash buffer to the column, full speed (≥ 1000 × g) Centrifuge for 30 seconds and discard the waste liquid.

⑦ Add 200 μL m-desnlphonation buffer, room temperature (20-30 ℃) for 15-20min, full speed (≥ 1000 × g) Centrifuge for 30s and discard the waste liquid.

⑧ Join 200 μ L m-wash buffer, fμLl speed (≥ 1000 × g) Centrifuge for 30 seconds and add 200 μ L m-wash buffer, full speed (≥ 1000 × g) Centrifuge for 30 seconds.

④ Add 10μL of m-elution buffer to the matrix of the column, put the column into a 1.5ml centrifuge tube, and centrifuge (≥ 1000 × g) 60s, and DNA was recovered. DNA was stored at - 20 ℃, and the amount of each PCR was 2-4 μL eluent.

4.2 PCR amplification

① Template: DNA extracted after methylation treatment of Roundup resistant transgenic rape and wild Brassica juncea forward and reverse backcross generation 1;

② According to the promoter (CaMV35s) and *PAT* gene sequence, methylation amplification is carried out in three stages. The designed primers are shown in Table S7;

③ See Table S8 for PCR reaction system;

④ The methylation PCR reaction conditions of promoter (CaMV35s) and *PAT* gene were as follows:

94℃ 5min

94℃ 30s

52.5℃ 30s 50Cycles

72℃ 2.5min

72℃ 10min

PCR products were detected by agarose gel electrophoresis.

4.3 Analysis method of transgenic methylation sites

In the DNA sequence, if cytosine (C) is methylated, the methylated cytosine will not be converted to uracil (U) under sulfite treatment, but still exists in the gene sequence as

cytosine (C). The transformants were sent to Shanghai biotechnology company for sequencing. With the aid of the analysis software Bioedit, the sequenced sequence was compared with the original sequence of the transgene (CaMV35s and *PAT* gene) to find the location and number of cytosine (C) in the measured sequence. Because there is certain error in the sequencing results, it is considered that the same methylation site appears twice or more in the three replicates of each single plant.

5.Testing of ABA content in leaves of progenies

Plants were selected in method described in 4.5.1. After 15 days of seed sowing, took the top first leaves from plants, immediately placed the weighed tissue (0.1g) in liquid nitrogen, and grinded thoroughly with a pestle.

5.1 Leave sample pretreatment

Added extraction solution (10% TCA) three times the volume of the sample and placed in the -20°C refrigerator for 24 hours. Centrifuged the sample for 1 hour at 4°C, 8000 rpm and collected the sediment. Added 0°C acetone of the same volume as the sediment and centrifuged at 4°C, 8000 rpm for 15 minutes. Discarded the supernatant and vacuum-dryed the sample for storage. Added 5mL of cracking solution (2.7g urea, 0.2g CHAPS and Dissolve thoroughly in ddH_2O and added volume to 5mL). Placed at room temperature for 30 minutes_then centrifuged at 4°C, 8000 rpm for 15 minutes.

5.2 ABA content testing

Collected the supernatant and solution was tested for ABA content by using an Abscisic acid ELISA detection kit. Compared the differences of ABA content in leaves between r-e and r-n-e plants.

6 Fitness analysis

6.1 Measurement method of fitness component

The fitness components measured in each vegetative growth period were plant height, stem diameter, number of primary branches, and dry biomass of single plant above ground. The fitness components measured during the reproductive growth period were the number of effective pod per plant, seed weight, pod length, and the number of full

seeds per pod. See Table S9 for specific measurement methods.

6.2 Calculation methods of fitness

Calculation method of relative fitness value of each fitness component: Taking wild Brassica juncea as the comparison standard "1", the ratio of each fitness component of the corresponding backcross progenies to the fitness component of wild Brassica juncea is the corresponding fitness value, and the total fitness value is the weighted average of the relative fitness values of each fitness component[38], In this test, the total fitness = ∑ (relative fitness value of each index) / 8.

Reference:

38. Mercer KL, Andow DA, Wyse DL, Shaw RG. Stress and domestication traits increase the relative fitness of crop-wild hybrids in sunflower. Ecol Lett. 2007, 10(5):383-93. DOI: 10.1111/j.1461-0248.2007.01029.x.
