# Supplementary material for: Stress Increases Ecological Risk of Glufosinate-Resistant Transgene Located on Alien Chromosomes in Hybrids Between Transgenic Brassica napus and Wild Brassica juncea
Source: Plants (Basel). 2025 Feb 13;14(4):572. doi: 10.3390/plants14040572 (PMC11859238; doi:10.3390/plants14040572)
Supplement: Supplementary file 1 [file plants-14-00572-s001.zip › Sup Tab.pdf]

Table S1 DNA PCR Primers for *PAT* gene

Primer name	Primer sequence	Application
PAT-F	5'- AGGACAGAGCCACAAACACCAC-3'	PCR amplification
PAT-R	5'- ACCAACATCATGCCATCCACCA-3'	PCR amplification

Table S2 The system of polymerase chain reaction for *PAT* gene

Reaction components	Concentration	Volume (μL)
Template	1.0 $\mu g \cdot \mu L^{-1}$	1.0
Primer P1	10.0 μM	1.0
Primer P2	10.0 μM	1.0
BU-Taq MIX	2×	7.0
ddH_2O	-	10.0
Total Volume	-	20.0

Table S3 Preparation for the reverse transcription master mix of *ROS1* genes in the backcross generation between wild *Brassica juncea* and transgenic glufosinate-resistant *B. napus*

Contents	Dosage of contents(/μL)
5×gDNA Eraser Buffer	2.0
gDNA Eraser	1.0
Total RNA	7.0
Total Volume	10.0

Table S4 Reaction for the reverse transcription master mix of *ROS1* in the backcross generation between wild *B. juncea* and transgenic glufosinate-resistant *B. napus*

Contents	Dosage of contents (μL)
Reaction solution after pretreatment in Table S3	10.0
PrimeScript RT Enzyme Mix I	1.0
RT Primer Mix	1.0

5×PrimeScript Buffer 2（for Real Time）	4.0
RNase Free dH_2O	4.0
Total Volume	20.0

Table S5 qPCR primers for *ROS1* and *HMG* genes in progenies of the first backcross generations between glufosinate-resistant transgenic *B. napus* and wild *B. juncea*

Primer names	Primer sequence
ROS1-F	5'-GAACTGGCTCAAGCGTAGAA-3';
ROS1-R	5'-CAAGACTCCCATGGTCTGTTAG-3';
HMG-F	:5'-GGTCGTCCTCCTAAGGCGAAAG-3';
HMG-R	: 5'-CTTCTTCGGCGGTCGTCCAC-3';

Table S6 qPCR amplification reaction system of *ROS1* genes in the backcross generation between wild *B. juncea* and transgenic glufosinate-resistant *B. napus*

Contents	Dosage of contents (μL)
10×Ex Taq Buffer	2.5
$MgCl_2$ （25 mM）	2
dNTP Mix （2.5 mM）	2
Primer-F （10 μM）	1
Primer-R （10 μM）	1
Ex Taq （5 U/μL）	0.2
cDNA	1
dH_2O	15.3
Total Volume	25

Table S7 The primers for DNA methylation PCR of transformant promoter (CaMV35S) and *PAT* gene

Primer name	Primer sequence	Length of amplification (bp)
MCa.P -F	5'- TTTTATGGAGTTAAAGATTTAAAT-3'	368
MCa.P -R	5'-AAAATCCATCTTTAAAACCACTATC -3'	
MCa.P1 -F	5'- GGAAAAGGAAGGTGGTTTTTATAA-3'	325
MCa.P1 -R	5'- AACCTAATCTCAACTAATCTCCTCTCC -3'	
MCa.P2 -F	5'- TTTTATTTGGAGAGGATAGGGTATT -3	237
MCa.P2 -R	5'- ACCTCAACAACCAACCAAAAATAT -3	
MCa.P3 -F	5'- TAGATATTTTTGGTTGGTTGTTGAG -3	351
MCa.P3 -R	5'- ACTAACAACTCAAAATCCCTTTACC -3	

Table S8 The system for PCR of transformant promoter (CaMV35S) and *PAT* gene

Reaction components	Concentration	Volume (μL)
DNA template	1.0 μg·μL^{-1}	2.0
P-F	15.0 μM	1.0
P-R	15.0 μM	1.0
Takara Taq Ex HS	5 U·μL^{-1}	0.4
Takara Taq E Buffer	10×	2.5
Mg^{2+}	25 mM	2.0
dNTP	2.5 mM	2.0
ddH2O	-	14.1
Total Volume	-	25.0

Table S9 Traits measured for relative fitness estimation at two growth stages

Vegetative growth traits	Reproductive growth traits
**Plant height:** Height from the base of the plant to the tip of the plant at maturity	**Seed weight:** The weight of seed/plant
**Stem diameter:** Main stem diameter of each plant measured at maturity	**Number of siliques/plant:** Number of siliques/plant at maturity
**Number of effective branches:** Number of branches with silique which carried seeds/plant	**Silique length:** Length of silique which were grown at lower part of plant measured at maturity stage (sample of at least 20 siliques per plant).
**Dry above-biomass:** Biomass measured after oven-dry of the final harvest as described by Simard et al. (2005)	**Seed number/silique:** Number of fully matured seeds in the silique measured its length
